# Mutant p53 in colon cancer

**DOI:** 10.1093/jmcb/mjy075

**Published:** 2018-11-29

**Authors:** Mizuho Nakayama, Masanobu Oshima

**Affiliations:** 1 Division of Genetics, Cancer Research Institute, Kanazawa University, Kanazawa, Japan; 2 WPI-Nano Life Science Institute, Kanazawa University, Kanazawa, Japan

**Keywords:** colon cancer, missense-type mutant p53, gain-of-function, mouse model, NF-κB, multistep tumorigenesis, organoids

## Abstract

The accumulation of genetic alterations in driver genes is responsible for the development and malignant progression of colorectal cancer. Comprehensive genome analyses have revealed the driver genes, including *APC*, *KRAS*, *TGFBR2*, and *TP53*, whose mutations are frequently found in human colorectal cancers. Among them, the p53 mutation is found in ~60% of colorectal cancers, and a majority of mutations are missense-type at ‘hot spots’, suggesting an oncogenic role of mutant p53 by ‘gain-of-function’ mechanisms. Mouse model studies have shown that one of these missense-type mutations, p53 R270H (corresponding to human R273H), causes submucosal invasion of intestinal tumors, while the loss of wild-type p53 has a limited effect on the invasion process. Furthermore, the same mutant p53 promotes metastasis when combined with Kras activation and TGF-β suppression. Importantly, either missense-type p53 mutation or loss of wild-type p53 induces NF-κB activation by a variety of mechanisms, such as increasing promoter accessibility by chromatin remodeling, which may contribute to progression to epithelial–mesenchymal transition. These results indicate that missense-type p53 mutations together with loss of wild-type p53 accelerate the late stage of colorectal cancer progression through the activation of both oncogenic and inflammatory pathways. Accordingly, the suppression of the mutant p53 function via the inhibition of nuclear accumulation is expected to be an effective strategy against malignant progression of colorectal cancer.

## Introduction

It has been suggested that the accumulation of driver gene mutations causes development and malignant progression of colorectal cancer. *TP53*/*Trp53* (human or mouse p53 gene) encoding tumor suppressor p53 is one of the important major driver genes for cancer development in various organs. In addition to the tumor-suppressing role of p53, it has been shown that some missense-type mutations of the p53 gene at the DNA binding domain induce acquisition of oncogenic functions. We therefore need to consider the role of p53 mutation in colorectal cancer development from two separate aspects: the ‘loss of wild-type p53 function’ and the ‘possible gain of oncogenic function of missense-type mutant p53’.

Recent genetic studies of human cancers as well as mouse model studies have revealed these complicated roles of p53 mutations in tumorigenesis. We herein review the role of mutant p53 (missense-type mutant p53 and loss of wild-type p53) highlighting what we have learned from human and mouse genetic studies.

## Multistep tumorigenesis of colorectal cancer

Colorectal cancer is the third-most common cancer and a leading cause of cancer-related death world-wide (Ferlay et al., 2013; Siegel et al., 2017). The 5-year survival rate drops significantly to about 14% for patients with metastasis (Howlader et al., 2017). It is therefore important to clarify the mechanisms underlying malignant progression process in order to develop a novel therapeutic strategy. Colorectal cancer begins as a benign adenomatous intestinal polyp, which progresses to advanced adenoma with high-grade dysplasia, invasive adenocarcinoma, and metastasis to distant organs. The concept of such gradual progression of colorectal cancer is known as ‘multistep tumorigenesis’, and each step is thought to be associated with specific genetic alterations in tumor suppressors or oncogenes (Fearon and Vogelstein, 1990; Markowitz and Bertagnolli, 2009; Fearon, 2011).

Recent genome-wide analyses have identified frequently mutated genes in human colorectal cancers, including *APC*, *KRAS*, *SMAD4*, and *TP53*, mutations of which are found in about 10–80% of non-hypermutated colorectal cancers (The Cancer Genome Atlas Network, 2012; Giannakis et al., 2016). Genetic alterations in these genes are thought to cause acquisition of stemness, increased cell proliferation, suppression of differentiation, and impaired genome DNA maintenance, which may contribute to ‘hallmarks of cancer’ (Hanahan and Weinberg, 2011) as major components of the cancer cell signaling pathways (Vogelstein et al., 2013). Furthermore, the modification of these pathways by the same genetic alterations is widely found in many types of cancers, suggesting that these are key driver pathways for oncogenesis, irrespective of tissue type (Sanchez-Vega et al., 2018).

The development of organoid culture techniques of normal intestinal stem cells has allowed us to validate the oncogenic role of driver mutations *in vitro* (Sato et al., 2009). It has been genetically demonstrated using human normal intestinal organoids that the simultaneous introduction of mutations in *APC*, *KRAS, PIK3CA*, *SMAD4*, and *TP53*, four or five major driver genes for colorectal cancer, induces epithelial cell transformation resulting in the development of transplantable tumors *in vivo* (Drost et al., 2015; Matano et al., 2015). These genetic experiments using organoids support the concept of multistep tumorigenesis of colon cancer, although additional epigenetic changes may be important for achieving a more advanced malignant phenotype (Matano et al., 2015).

## Mutations of *TP53* in human colorectal cancer

Germline mutations in *TP53* are responsible for Li-Fraumeni syndrome, a familial cancer syndrome of diverse tumors, indicating a tumor suppressor role of p53 in a variety of tissues (Malkin et al., 1990; Srivastava et al., 1990). Consistently, the most frequently mutated gene in the Pan-Cancer cohort is *TP53* (42% of all samples examined) (Kandoth et al., 2013). In non-hypermutated colorectal cancers, the mutation rate of *TP53* is about 55%–60%, which is the second-most frequent mutation observed in colorectal cancer (*APC* mutation is found >80%), whereas the mutation rate of *TP53* is significantly decreased to ~20% in hypermutated colorectal cancers (The Cancer Genome Atlas Network, 2012; Kandoth et al., 2013; Giannakis et al., 2016).

Moreover, *TP53* mutations have been associated with a poor prognosis in several types of cancers, including colorectal cancers (Olivier et al., 2010). Consistently, when advanced colorectal cancer patients who suffered metastasis were examined, the *TP53* mutation rate increased to 80% (Brannon et al., 2014). On the other hand, *TP53* mutations are not common in benign colonic adenomatous polyps (Borras et al., 2016), and its alteration frequencies are about 15%–30% (Baker et al., 1990; Hao et al., 2002). These results support the hypothesis that *TP53* mutations play a role in the late stage of tumorigenesis, such as progression step of adenomas to invasive adenocarcinomas or metastasis to distant organs.

Cellular stress, like DNA damage, telomere erosion, hypoxia, and nutrient depletion, as well as oncogenic signaling, activates p53, leading to the promotion of cell-cycle arrest, senescence, and apoptosis depending on the extent and context of the stress (Vousden and Lu, 2002; Vousden and Prives, 2009). These distinct stress responses are regulated by subsets of p53 target molecules, including p21, Puma, Tiger, and PAI-1, which respond to different p53-activating stimuli (Beckerman and Prives, 2010). The loss of p53 transcriptional activity that causes the suppression of these p53 target induction is thought to be a major mechanism by which *TP53* loss-of-function mutations promote tumorigenesis in various organs including colon.

## Missense-type mutant p53 for cancer development

It has been shown that a majority (~74%) of *TP53* somatic mutations in cancer cells are missense-type mutations with amino acid substitutions in the DNA binding domain. There are several ‘hot spot’ residues (R175, G245, R248, R249, R273, and R282). Since p53 normally functions as a tetramer, such mutant p53 may inhibit the role of remaining wild-type p53 by a ‘dominant-negative’ mechanism. It has been shown that the expression of mutant p53 R172H (corresponding to human R175H) in a mouse model attenuates the therapeutic effect induced by the restoration of wild-type p53 (Wang et al., 2011). However, accumulating evidence has shown that *TP53* mutations at these hot spots can induce acquisition of an oncogenic function via a ‘gain-of-function’ mechanism (Brosh and Rotter, 2009; Muller and Vousden, 2014), although each mutant p53 can have distinct effects on tumorigenesis (Mello and Attardi, 2013; Muller and Vousden, 2014). Moreover, these missense-type *TP53* mutations are significantly and positively correlated with a poor survival in colorectal cancer patients, suggesting a role of gain-of-function by p53 mutation in malignant progression step (Cooks et al., 2018). Notably, in the non-hypermutated human colon cancer, about 80% of tumors that carry missense mutations lost wild-type *TP53* genes via loss of heterozygosity (LOH) (The Cancer Genome Atlas Network, 2012). Taken together, these results suggest that the presence of missense-type mutant p53 at hot spots can promote progression of colon tumors from adenoma to adenocarcinoma by gain-of-function mechanism, and the additional loss of wild-type p53 by LOH induces a more advanced phenotype at a later stage of tumorigenesis.

Mouse model studies have provided genetic evidence of the gain-of-function mechanism by two major missense-type mutant p53 in tumorigenesis. Genetically engineered mouse models that express *Trp53 R172H* or *R270H* mutations (corresponding to the human *TP53 R175H* or *R273H*, respectively) developed spontaneous adenocarcinomas in the intestine and lung with the nuclear accumulation of p53, findings not noted in *Trp53^Null^* (deleted) mutant mice (Lang et al., 2004; Olive et al., 2004). Several possible mechanisms for tumorigenesis by the missense-type mutant p53 proteins have been reported. For example, mutant p53 protein drives invasiveness and the scattered migration of cancer cells by promoting Rub coupling protein (RCP)-recycling of integrin and the receptor for hepatocyte growth factor (HGF), MET, thereby resulting in the activation of EGFR/integrin and HGF signaling, respectively (Muller et al., 2009, 2013). Furthermore, mutant p53 promotes cancer metastasis by binding p63 and p73, which accelerates TGF-β-induced metastasis and PDGF receptor β signaling, respectively (Adorno et al., 2009; Weissmueller et al., 2014). It has also been shown that mutant p53 increases the stem cell pool of mammary epithelial cells, which causes increased susceptibility for tumorigenesis (Lu et al., 2013).

Recently, epigenetic mechanisms induced by mutant p53 protein for tumorigenesis have been demonstrated, which explains the transcriptional activation of myriad genes, including oncogenes, by the expression of mutant p53 (Freed-Pastor and Prives, 2012). Several missense-type mutations of p53, including R175H and R273H have shown to induce global transcriptional shift by epigenetic switching of promoters through interaction with the SWI/SNF chromatin remodeling complex or the induction of MLL1, MLL2, and MOZ to modify histone methylation and acetylation (Pfister et al., 2015; Prives and Lowe, 2015; Zhu et al., 2015). A mouse model study using ‘sleeping beauty’ transposon also indicated that the mutation of *Arid1a*, a component of SWI/SNF, was enriched by transposons in *in vivo* intestinal tumors when the mouse carried heterozygous *Trp53* R172H (human R175H) mutation, suggesting an additive effect of mutations in p53 and the SWI/SNF complex for intestinal tumorigenesis (Takeda et al., 2015). These results suggest that the presence of missense-type mutant p53 at hot spots can induce transcriptome shift via the modification of the chromatin status of gene promoters in colorectal cancer cells.

## Loss of wild-type p53 in intestinal tumor mouse model

*APC* gene mutation is a gate keeper event for colorectal cancer development that activates canonical Wnt signaling. In normal intestinal stem cells, Wnt ligands from stromal cells stimulate Frizzled receptors, resulting in the destruction of the APC, β-catenin, and GSK3β complex that is required for β-catenin degradation. Wnt signaling thus induces β-catenin stabilization and translocation to the nucleus, forming a complex with TCF, and then β-catenin functions as a transcription factor to induce Wnt-target genes, which plays an important role in stem cell maintenance (Nusse and Clevers, 2017). Accordingly, the *APC* mutation causes intestinal tumorigenesis via the constitutive activation of Wnt signaling in epithelial cells.

Chemically induced or genetically engineered heterozygous *Apc* mutant models (*Apc*^*Min*^ and *Apc*^*Δ716*^ mice, respectively) develop multiple intestinal polyps upon the somatic loss of wild-type *Apc* gene by LOH, which results in constitutive Wnt signaling activation (Moser et al., 1990; Oshima et al., 1995). To examine the effect of the loss of wild-type p53 on intestinal tumorigenesis, several compound mutant mice were generated by crossing (Jackstadt and Sansom, 2016). The introduction of the Null allele for *Trp53* in *Apc*^*Min*^ mice did not cause any progression of intestinal adenomas (Clarke et al., 1995; Fazeli et al., 1997). These studies used mice with a heterogenous genetic background of C57BL/6 and 129 strains. Interestingly, when *Apc*^*Min*^*Trp53^−/−^* mice were examined with a C57BL/6 inbred background, local submucosal invasion was found at a low frequency (Halberg et al., 2000). Taken together, these results suggest that the loss of wild-type p53 in the absence of mutant p53 might contribute to the invasion of benign tumors, although it is not sufficient to drive the process, and modifier gene alteration depending on genetic background may be required.

## Missense-type mutant *Trp53* in intestinal tumor mouse model

**Figure 1:**
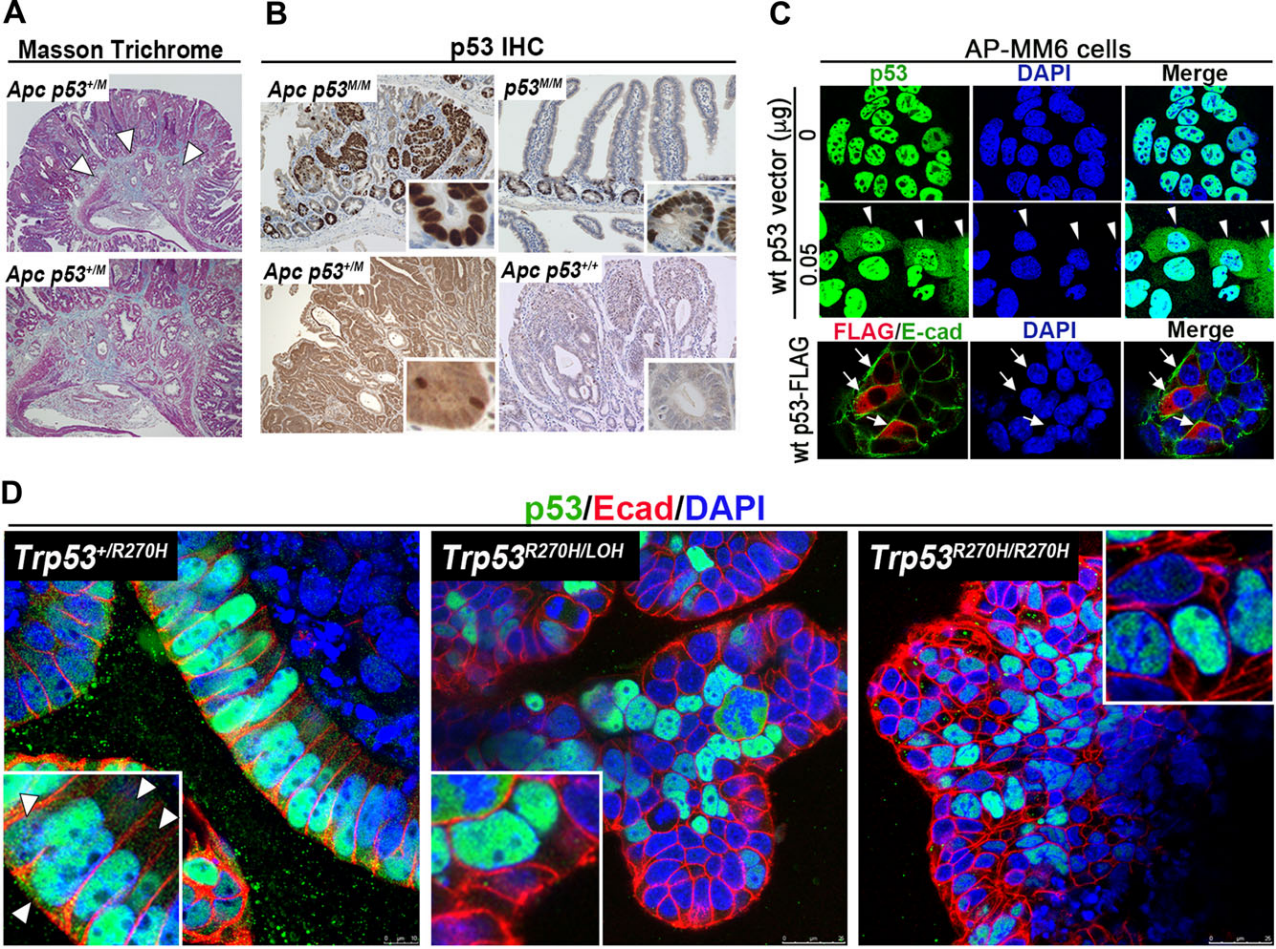
Submucosal invasion and the nuclear accumulation of mutant p53. (**A**) Submucosal invasion of *Apc*^*Δ716*^*Trp53*^*+/R270H*^ (*Apc Trp53*^*+/M*^) mouse intestinal tumors shown by Masson Trichrome staining. The arrowheads indicate the invasion area (enlarged image at the bottom). (**B**) p53 immunohistochemistry of intestinal tumors. The nuclear accumulation is detected in *Apc*^*Δ716*^*Trp53*^*R270H/R270H*^ (*Apc Trp53*^*M/M*^, *Trp53* homozygous mutant) tumor cells (top left), while it is stabilized but distributed to the cytoplasm in *Apc*^*Δ716*^*Trp53*^*+/R270H*^ (*Apc Trp53*^*+/M*^, *Trp53* heterozygous mutant) tumor cells (bottom left). p53 is not detected in *Apc*^*Δ716*^*Trp53*^*+/+*^ tumors (bottom right). Notably, the nuclear accumulation of mutant p53 R270H is also detected in normal intestinal cryptic cells (top right). (**C**) p53 is detected in the nuclei of AP-MM6 cells (derived from *Apc*^*Δ716*^*Trp53*^*R270H/R270H*^ intestinal tumors) (top). Transfection of wild-type p53 into AP-MM6 cells resulted in partial cytoplasmic distribution of p53 (middle, arrowheads; bottom, arrows). (**D**) Confocal images of immunostaining for p53 (green) and E-cadherin (red) with DAPI staining (blue) of *Apc*^*Δ716*^*Kras*^*G12D*^*Tgfbr2*^*−*/*−*^*Trp53*^*R270H*^ mouse tumor-derived organoids. The *Trp53* genotypes of tumor cells are *Trp53*^*+/R270H*^ (left), *Trp53*^*R270H/LOH*^ (center), and *Trp53*^*R270H/R270H*^ (right). Note that mutant p53 is exclusively detected in nuclei when wild-type p53 is not expressed (center and right), while it is also partially distributed to the cytoplasm of *Trp53* heterozygous tumor cells (left, arrowheads). (**A**–**C**) Modified from Nakayama et al. (2017).

In contrast to the Null mutation, the introduction of heterozygous mutations of *Trp53 R172H* or *R270H* in *Apc*^*Min*^ or *Apc*^*Δ716*^ mice, respectively, induced submucosal invasion of intestinal tumors (Figure 1A) (Muller et al., 2009; Nakayama et al., 2017). The incidence of invasion was significantly higher in *Apc*^*Δ716*^*Trp53*^+/*R270H*^ mice than in *Apc*^*Δ716*^*Trp53*^*Null*^ mice (9% vs. 0% of small tumors <3 mm, and 62% vs. 16% of large tumors ≥3 mm), and high-grade dysplasia was found in the invading tumors with *Trp53 R270H* mutations (Nakayama et al., 2017).

As discussed above, the loss of wild-type p53 by LOH in addition to the missense-type mutation is thought to be important for malignant progression of cancers. However, we noted no loss of wild-type *Trp53* at the invasion front of *Apc*^*Δ716*^*Trp53*^+/*R270H*^ mice. These results suggest that missense-type mutant p53 at codon R172 and R270 (human codon R175 and R273, respectively) is a key factor inducing submucosal invasion of intestinal tumors by gain-of-function mechanism, and the loss of wild-type p53 by LOH may not be required for this process.

## The nuclear accumulation of mutant p53 and its possible regulation mechanism

In *Apc*^*Δ716*^*Trp53*^*R270H/R270H*^ mouse intestinal tumors (*Trp53 R270H* homozygous mutant), the clear nuclear accumulation of p53 was detected by immunohistochemistry. However, in the *Apc*^*Δ716*^*Trp53*^*+/R270H*^ mice (*Trp53^+/R270H^* heterozygous mutant), p53 was detected but distributed to the cytoplasm (Figure 1B) (Nakayama et al., 2017). These results suggest that wild-type p53 interferes with the nuclear accumulation of mutant p53 in the heterozygous tumor cells.

**Figure 2:**
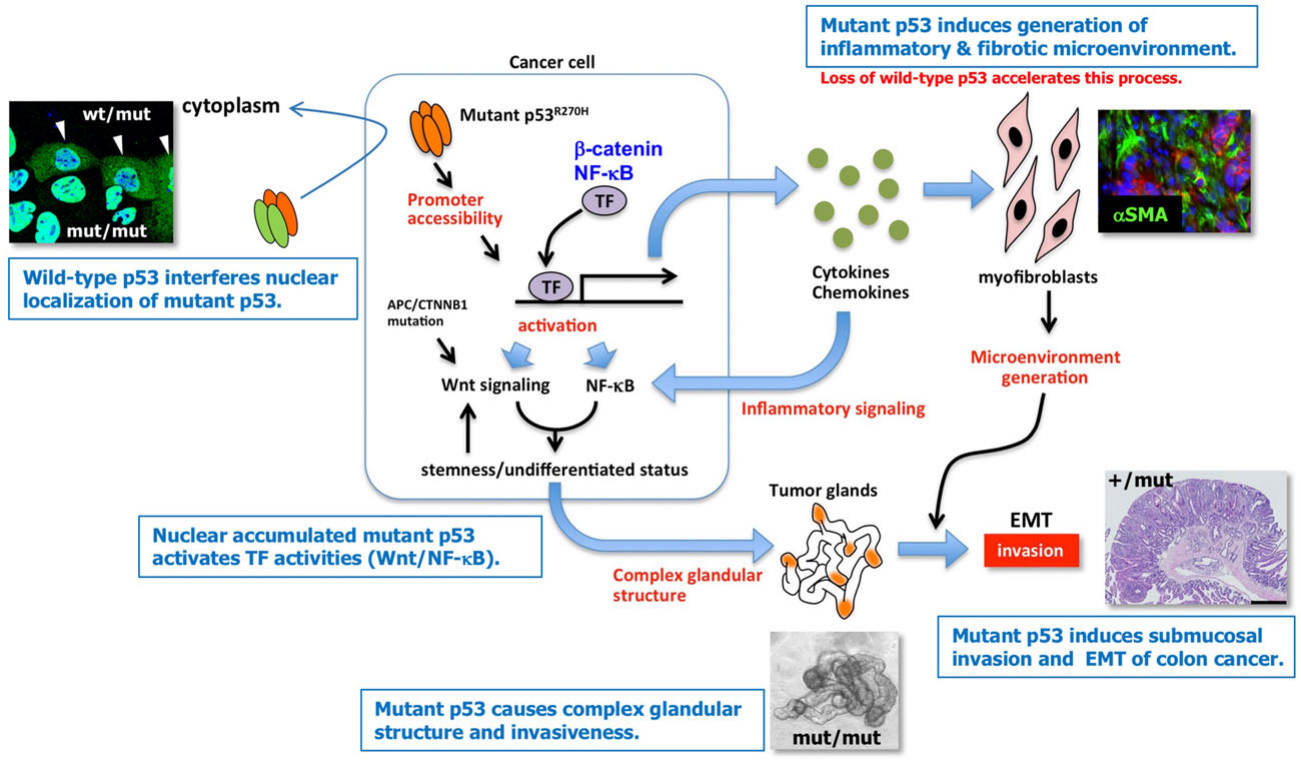
Possible mechanism by which missense-type mutant p53 affects the malignant progression of colorectal cancer cells (explanation in counterclockwise). Loss of wild-type p53 may contribute to the nuclear accumulation of mutant p53; mutant p53 activates transcription factors (TFs), including Wnt/β-catenin and NF-κB, by chromatin remodeling; mutant p53 induces the glandular structure of tumor organoids, possibly caused by Wnt hyper-activation; mutant p53 induces submucosal invasion, with loss of wild-type p53 not necessary for this process; mutant p53 induces the generation of an inflammatory microenvironment via fibrotic reactions, consisting of αSMA-positive myofibroblasts, which may be accelerated by loss of wild-type p53 (Modified from Nakayama et al., 2017).

This hypothesis was further tested in cell culture experiments. We established a cell line from *Apc*^*Δ716*^*Trp53*^*R270H/R270H*^ mouse intestinal tumors (AP-MM6 cells), which show the clear nuclear accumulation of mutant p53. Importantly, transfection of wild-type p53 expression vector to AP-MM6 cells resulted in the partial cytoplasmic distribution of p53 (Figure 1C). We further found that p53 is clearly accumulated in the nucleus in *Trp53*^*R270H/R270H*^ and *Trp53*^*R270H/LOH*^ tumor organoids, while cytoplasmic distribution was also found in *Trp53*^*+/R270H*^ organoid cells (Figure 1D). It is therefore possible that the chimeric tetramer complex consisting of mutant p53 and wild-type p53 cannot remain in the nuclei (Figure 2). On the other hand, it has been reported that the loss of wild-type p53 is an important prerequisite for the stabilization of mutant p53 in breast cancer and sarcomas, suggesting that chimeric p53 tetramers are not stable in these cells (Alexandrova et al., 2017). We have not examined how the chimeric p53 tetramers are stabilized in the cytoplasm of intestinal tumor cells, however, it is possible that stability of chimeric p53 complex varies according to the tissue type.

Interestingly, it has also been reported that mutant p53 R172H is stable only in tumor cells but not in normal tissues (Terzian et al., 2008). However, we found that p53 R270H proteins are detected in the tumor cells as well as in the normal intestinal cryptic cells of *Apc*^*Δ716*^*Trp53*^*R270H/R270H*^ mice (Figure 1B). Consistently, p53 R172H is detected in the intestinal crypt of wild-type mice (Goh et al., 2015). Mutant p53 R172H protein expressed in non-tumorous cells has been shown to be regulated by Mdm2, an E3 ubiquitin ligase for p53, resulting in degradation through the proteasome pathway in a manner similar to wild-type p53. Thus, stabilization is not a result of mutation and is therefore a prerequisite for the gain-of-function of missense-type mutant p53.

Intriguingly, we found that p53 is accumulated in the nucleus in a subset of tumor cells at the invasion front of *Apc*^*Δ716*^*Trp53*^*+/R270H*^ mice (*Trp53^+/R270H^* heterozygous mutant) (Nakayama et al., 2017). Taken together, these findings suggest that the microenvironment of the invasion front as well as normal intestinal crypts contributes to the stabilization and nuclear localization of mutant p53 by exogenous stimulation. These results suggest that missense-type mutant p53 drives the submucosal invasion of the primary intestinal tumors when the microenvironment supports the nuclear accumulation of p53, although it still remains to be elucidated whether the nuclear accumulation of mutant p53 is indeed required for malignant progression.

## Missense-type mutant *p53* for advanced cancer phenotypes

We further examined the biological characteristics of tumor organoids derived from *Apc*^*Δ716*^*Trp53*^*R270H/R270H*^ mice and *Apc*^*Δ716*^*Trp53*^*Null*^ mice. In the homozygous *Trp53*^*R270H/R270H*^ tumor organoids, mutant p53 is constitutively localized in nuclei, as shown above. The morphology of organoids from *Apc*^*Δ716*^ mice shows a round cystic structure because of the uniformly activated Wnt signaling and lack of a differentiation gradient (Sato et al., 2011); this phenotype was not changed when the wild-type p53 was lost. In contrast, *Trp53*^*R270H/R270H*^ homozygous tumor organoids formed a complexed glandular structure that was strikingly different from the *Trp53^Null^* mutant organoids (Figure 2). This glandular structure of tumor organoids was associated with the significant increase of Wnt activation level and acquisition of invasiveness, while *Apc*^*Δ716*^*Trp53*^*Null*^ organoids showed limited invasion ability. It remains to be investigated whether organoid culture conditions affect the acquisition of such tumor characteristics; however, the results suggest that mutant p53 induces undifferentiated status by Wnt signaling activation. These malignant phenotypes associated with the expression of mutant p53 R270H were significantly suppressed by the treatment of organoid cells with suberoylanilide hydroxamic acid (SAHA), a histone deacetylase (HDAC) inhibitor that induces the degradation of mutant p53 through the Mdm2-mediated ubiquitin proteasome system (Li et al., 2011; Alexandrova et al., 2015).

In addition, the subcutaneous transplantation of *Apc*^*Δ716*^*Trp53*^*R270H/R270H*^ tumor-derived organoids caused more aggressive phenotypes than *Apc*^*Δ716*^*Trp53*^*Null*^ tumor-derived organoid did, such as epithelial–mesenchymal transition (EMT)-like morphology with increased branching and the generation of a desmoplastic (fibrotic) microenvironment consisting of activated myofibroblasts (Figure 2). Increased branching of tumor glands was also evident in human colon cancers with *TP53* mutations around codon 273. It has been suggested that a desmoplastic microenvironment is important for the induction of the EMT phenotype of cancer cells (Lambert et al., 2017). Furthermore, colon cancers with fibrotic stroma are classified as ‘consensus molecular subtypes’ (CMS) type 4, which is associated with metastasis and a poor prognosis (Guinney et al., 2015; Dienstmann et al., 2017). Accordingly, it is possible that the missense-type mutant p53 promotes malignant progression of colorectal cancer by the induction of a complex glandular structure, acquisition of invasiveness, and induction of fibrotic microenvironment generation (Figure 2).

## Combination of mutant p53 with other driver mutations in the course of multistep tumorigenesis

Recently, colon cancer mouse models that recapitulate genetic alterations of human cancers were constructed by simultaneous introduction of *Apc* mutation, *Kras*^*G12D*^ mutation, and *Trp53* loss or *Trp53* missense-type mutation in organoid cells and orthotopic transplantation of these engineered organoids to mice, thereby causing the development of invasive colonic adenocarcinoma and lymph node or liver metastasis (O’Rourke et al., 2017; Roper et al., 2017; Fumagalli et al., 2018).

**Figure 3:**
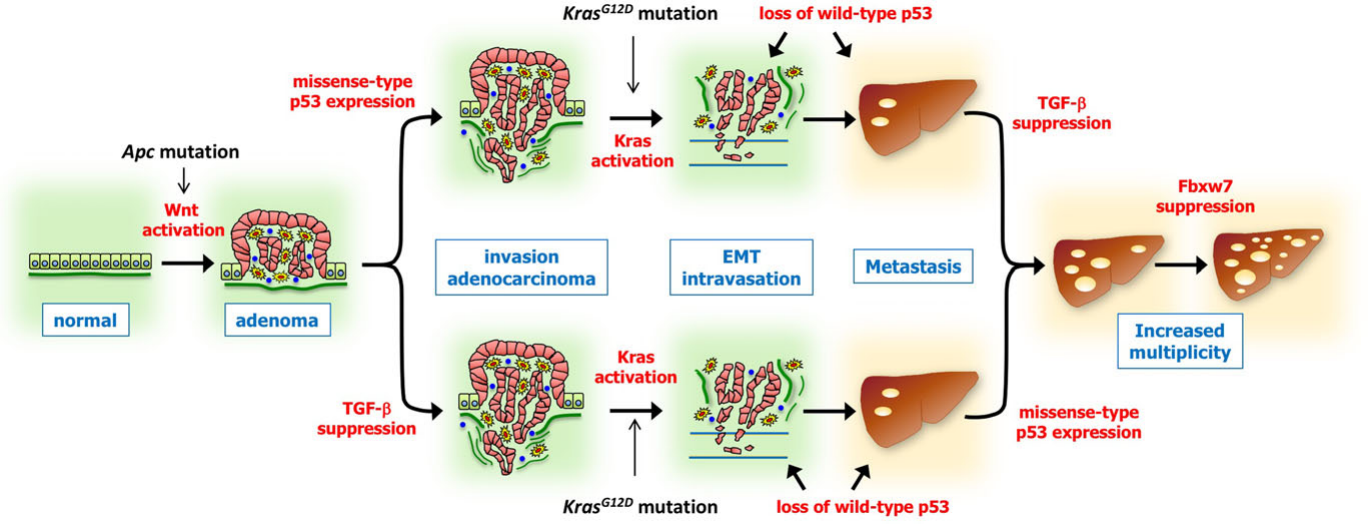
Multistep tumorigenesis of colorectal cancer development and malignant progression based on the results of multi-crossing mouse genetic experiments (Sakai et al., 2018). The expression of missense-type p53 mutation or TGF-β suppression causes submucosal invasion, and additional *Kras* mutation activation induces more malignant phenotypes, like EMT and metastasis. The loss of wild-type p53 supports the later stage of malignant progression. Combinations of four and five driver mutations, including missense-type p53 mutation, are required for an advanced metastatic ability.

To further examine the role of missense-type mutant p53 in combination with other driver mutations, we constructed mouse models carrying all possible combinations of the driver mutations of *Apc*^*Δ716*^, *Kras*^*G12D*^, *Tgfbr2*^*−*/*−*^, *Trp53*^*+/R270H*^, and *Fbxw7*^*−*/*−*^ (Oshima et al., 2015; Nakayama et al., 2017; Sakai et al., 2018). Among the generated mouse genotypes, double mutations of *Apc*^*Δ716*^*Tgfbr2*^*−*/*−*^ and *Apc*^*Δ716*^*Trp53*^*+/R270H*^ resulted in the development of invasive adenocarcinoma in the intestine, suggesting that Wnt signaling activation in combination with TGF-β suppression or the expression of mutant p53 R270H induces submucosal invasion (Figure 3), while *Apc*^*Δ716*^*Kras*^*G12D*^ combination is not sufficient for generating an invasive phenotype.

We noted that mutational combinations of *Apc*^*Δ716*^*Tgfbr2*^*−*/*−*^ and *Apc*^*Δ716*^*Trp53*^*+/R270H*^ are not sufficient for generating more advanced malignant phenotypes. Importantly, an additional *Kras* mutation, i.e. combinations of *Apc*^*Δ716*^*Kras*^*G12D*^*Tgfbr2*^*−*/*−*^ or *Apc*^*Δ716*^*Kras*^*G12D*^*Trp53*^*+/R270H*^ triple mutations, induced advanced phenotypes like intravasation to capillary vessels and EMT-like morphology in the invasion areas (Figure 3). Consistently, transplantation experiments using established tumor organoids indicated that triple mutations of *Apc*^*Δ716*^*Kras*^*G12D*^*Tgfbr2*^*−*/*−*^ and *Apc*^*Δ716*^*Kras*^*G12D*^*Trp53*^*+/R270H*^ are minimum cores for liver metastasis, but other double mutations or even triple mutation of *Apc*^*Δ716*^*Trp53*^*+/R270H*^*Tgfbr2*^*−*/*−*^ are not sufficient for the induction of metastasis, suggesting that a *Kras* mutation in addition to Wnt activation and TGF-β suppression or p53 mutation is critical for metastasis (Figure 3).

Furthermore, among metastatic tumor organoids, we found that the rate of multiplicity of metastasis was significantly higher in *Apc*^*Δ716*^*Kras*^*G12D*^*Tgfbr2*^*−*/*−*^*Trp53*^*+/R270H*^ cells than in triple mutant organoids, and quintuple *Apc*^*Δ716*^*Kras*^*G12D*^*Tgfbr2*^*−*/*−*^*Trp53*^*+/R270H*^*Fbxw7*^*−*/*−*^ cells showed the highest multiplicity. The gene expression profiles were also evolutionally changed accordingly (Supplementary Figure S1). These results suggest that the expression of mutant p53 and the suppression of the TGF-β pathway may share oncogenic pathways that induce invasion, EMT, and metastasis; and the combination of missense-type mutant p53 and TGF-β suppression synergistically accelerates metastasis when combined with Kras activation and *Fbxw7* disruption.

## Mutant p53 for inflammatory pathway activation

Accumulating epidemiological and genetic results have suggested that inflammatory responses promote colorectal cancer development, making this a potential preventive or therapeutic target (Grivennikov et al., 2010; Wang and Dubois, 2010). In the tumor microenvironment, macrophages are infiltrated (they are called ‘tumor-associated macrophages’) and activated, expressing CSF-1 and CCL2, which may promote intravasation and metastasis (Qian and Pollard, 2010; Kitamura et al., 2015). Macrophage-expressing cytokines can stimulate tumor cells, activating the transcription factors NF-κB and Stat3 that link inflammation and cancer (He and Karin, 2011). In chemically induced colitis-associated colon cancer models, it has been shown that NF-κB activation in both stromal cells and tumor cells is important for tumorigenesis (Greten et al., 2004). Furthermore, NF-κB activation together with Wnt signaling activation in differentiated epithelial cells has shown to induce dedifferentiation of intestinal epithelial cells and the acquisition of stem cell properties (Schwitalla et al., 2013a).

However, the mechanism underlying NF-κB activation in tumor tissues has not yet been fully elucidated. Several studies, including one from our group, have shown the possible role of missense-type mutant p53 in NF-κB activation in intestinal tumors. Schwitalla et al. (2013b) showed that the loss of wild-type p53 exacerbated chemically induced colitis-associated colon tumorigenesis with the induction of EMT phenotypes. Mechanistically, they found that *Trp53* disruption suppresses the expression of the tight junction molecules occludin and claudin, which causes a defect in the barrier function of the epithelia. Subsequently, bacterial products like lipopolysaccharide may infiltrate into the lamina propria, leading to the activation of residential macrophages, which can further activate NF-κB in both tumor cells and stromal cells. It has also been shown that increased permeabilization of tumor cell junctions results in the activation of macrophages by bacterial infiltration, which induces IL-23- and Th17-mediated intestinal tumor growth (Grivennikov et al., 2012).

Cooks et al. (2013) showed that the expression of mutant p53 R172H is associated with persistent inflammatory responses in chemically induced ulcerative colitis in mouse model, which results in increased susceptibility to colitis-associated colon cancer development. TNF-α signaling activates NF-κB in intestinal epithelia to induce target genes. When mutant p53 is expressed, NF-κB binding to the target gene promoter is significantly prolonged, leading to the persistent induction of inflammatory cytokine expression. In human colitis-associated colon cancers, *TP53* mutations are the most common founder mutations and are also found in non-tumorous inflamed mucosa, possibly caused by reactive oxygen species generation (Hussain et al., 2000; Leedham et al., 2009; Thorsteinsdottir et al., 2011). Accordingly, it is possible that the expression of missense-type mutant p53 in ulcerative colitis is prone to colon cancer development through induction of inflammatory responses.

We performed RNA sequencing of tumor organoids from *Apc*^*Δ716*^*Trp53*^*+/+*^, *Apc*^*Δ716*^*Trp53*^*Null*^, and *Apc*^*Δ716*^*Trp53*^*R270H/R270H*^ mice (*Trp53* genotypes are different) and found ~350 genes that are specifically upregulated in p53 R270H-expressing tumors (Nakayama et al., 2017). An ingenuity pathway analysis using these genes revealed that the Wnt signaling pathway and NF-κB pathway are significantly activated via a mutant p53 R270H expression-dependent mechanism. We also found that promoter accessibility is increased significantly in the *Apc*^*Δ716*^*Trp53*^*R270H/R270H*^ tumor cells, suggesting that the chromatin structures of gene promoters are opened by mutant p53, which may result in the wide activation of the Wnt/β-catenin and NF-κB pathways (Figure 2). The mechanism by which mutant p53 modifies chromatin is consistent with previous findings (Pfister et al., 2015; Prives and Lowe, 2015; Zhu et al., 2015). The activation of both the Wnt signaling and NF-κB pathways by mutant p53 may cause increased stemness or an undifferentiated status of tumor cells (Schwitalla et al., 2013a) (Figure 2).

Other mechanisms of mutant p53 for NF-κB activation have also been reported. For example, mutant p53 activates TNF-α-induced NF-κB activation in cancer cells by binding to tumor suppressor DAB2IP (Di Minin et al., 2014). Moreover, colon cancer cells expressing mutant p53 selectively shed miR-1246-enriched exosomes, which reprograms the surrounding macrophages to express the immunosuppressive cytokines TGF-β and IL-10 (Cooks et al., 2018). Taken together, these results suggest that the expression of missense-type mutant p53 as well as loss of wild-type p53 play a role in the modulation of the inflammatory and immune microenvironment in cancer tissues through a variety of mechanisms, which significantly contributes to the induction of EMT and metastasis of colorectal cancer.

## Loss of wild-type *Trp53* for malignant progression of intestinal tumors in the absence of missense-type mutations

As discussed above, loss of wild-type p53 in addition to missense-type p53 mutation is found in later stages of colon cancer progression. However, a copy number decrease of *TP53* without missense-type mutation was also found in ~15% of non-hypermutated colorectal cancer (The Cancer Genome Atlas Network, 2012), suggesting that the partial suppression of the wild-type p53 function may contribute to malignant progression in these patients.

A computational network-based analysis revealed that Notch activation and deletion of wild-type p53 exert a synergistic effect on the activation of EMT-like processes of colorectal cancer (Chanrion et al., 2014). Although genetic alterations in the Notch pathway are not common in colorectal cancer, Notch activation is an important cancer driver pathway (Vogelstein et al., 2013; Sanchez-Vega et al., 2018). Furthermore, Notch signaling is activated in colon cancer stem cells, which may be important for the survival and self-renewal characteristics of tumor cells (Fre et al., 2009; Sikandar et al., 2010). Mechanistically, Notch signaling induces the expression of EMT factors, such as Zeb1, Snail, Slug, and Twist, which is suppressed at the translational level by microRNAs that are transcriptionally regulated by p53 family genes. Accordingly, wild-type p53 suppresses Notch activation-associated invasive adenocarcinoma development, and thus, the loss of wild-type p53 may accelerate Notch-induced progression to invasive adenocarcinoma with EMT phenotypes (Chanrion et al., 2014).

A similar mechanism has been shown in different mouse model study. *BRAF* is one of the major driver genes of colorectal cancers, and the most frequent somatic mutation is a point mutation at codon 600 (*BRAF*^*V600E*^), which results in a several hundred-fold increase in kinase activity compared with wild-type (Davies et al., 2002). The expression of *Braf*^*V637E*^ in mouse intestinal epithelial cells (corresponding to human *BRAF*^*V600E*^) induced hyperplasia and adenoma development, and wild-type p53 was activated in the high-grade dysplastic areas of tumors (Rad et al., 2013). Notably, the loss of wild-type p53 in this model induced invasive adenocarcinoma development and distant metastasis.

Accordingly, these results suggest that tumor suppressor p53 is activated during malignant progression of colorectal tumorigenesis, which suppresses Notch activation-induced EMT by microRNA regulation and BRAF activation-induced malignant phenotype; thus, the loss of wild-type p53 accelerates malignant progression (Figure 3).

## Conclusions and perspective

Recent progress in cancer modeling based on genome information and innovative technologies, such as organoid cultures, have allowed us to generate mouse models that recapitulate the process of malignant progression and metastasis of human colorectal cancer. Using these models, it has been shown that the presence of missense-type mutant p53 at hot spots, such as p53 R270H (human R273H), plays a role in submucosal invasion, the induction of EMT, and metastasis when combined with other driver mutations. Although several oncogenic mechanisms induced by mutant p53 have been reported, changes in the chromatin structure are possibly important for gain-of-function mechanism induced by mutant p53 that can induce global transcriptome shift. In colorectal cancer cells, Wnt signaling is activated by *APC* mutations, and NF-κB is also activated by the inflammatory microenvironment. Furthermore, cooperation of the Wnt signaling and the NF-κB pathways have shown to induce stemness and tumorigenicity. Thus, the simultaneous hyperactivation of the Wnt and NF-κB pathways in colorectal cancer cells caused by changing the chromatin status of the target gene promoter is one of the important oncogenic mechanisms by missense-type mutant p53 (Figure 2). This mechanism also suggests that the expression of mutant p53 is important for the tumor-initiating ability of colorectal cancer stem cells.

Importantly, the nuclear accumulation appears to be required for the oncogenic function of mutant p53. Although loss of wild-type p53 may cause stabilization of mutant p53, it has also been shown that even after the loss of wild-type p53, mutant p53 stabilization and nuclear localization are still regulated by exogenous signaling. Accordingly, further studies will be required to understand the mechanism underlying mutant p53 stabilization and subcellular localization by microenvironment-derived signaling. However, such signaling will be an ideal target for therapies to prevent colorectal cancer progression.

## Supplementary Material

Supplementary Data
