# Supplementary figures and images for: Mutant p53 in colon cancer
Source: J Mol Cell Biol. 2018 Nov 29;11(4):267–76. doi: 10.1093/jmcb/mjy075 (PMC6487790; doi:10.1093/jmcb/mjy075)

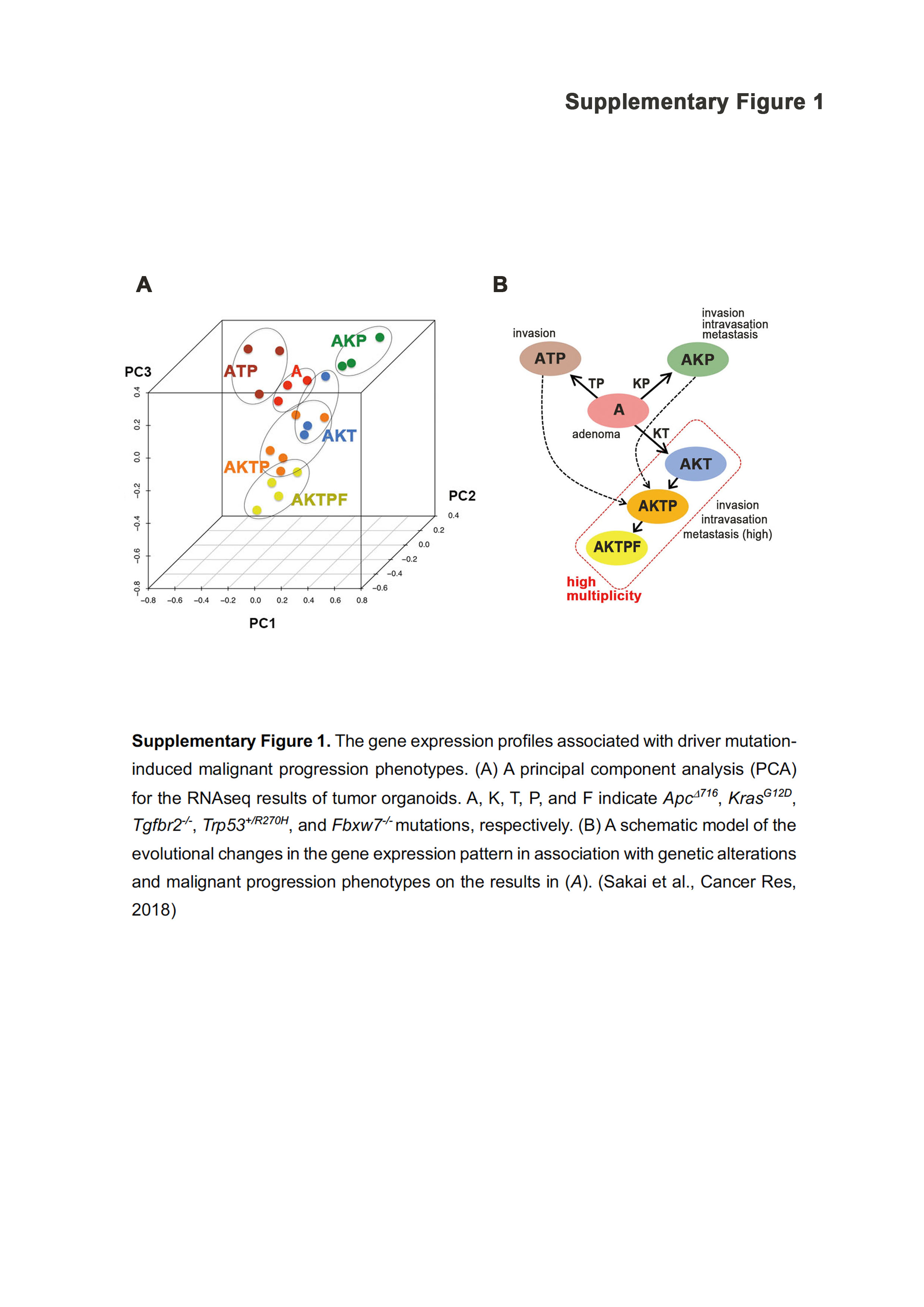

Supplement: Supplementary Data [file mjy075_supplementaryfig1.jpeg]
